# Is there an impact of a video-based patient informed consent in elective hand surgery?

**DOI:** 10.1007/s00402-024-05291-9

**Published:** 2024-06-08

**Authors:** Justus Osterloh, Wibke Müller-Seubert, Aijia Cai, Andreas Arkudas, Raymund R. E. Horch

**Affiliations:** Department of Plastic and Hand Surgery and Laboratory for Tissue Engineering and Regenerative Medicine, University Hospital Erlangen, Friedrich-Alexander University Erlangen-Nürnberg (FAU), Erlangen, Germany

**Keywords:** Informed consent, Hand surgery, Multimedia, Patient care

## Abstract

**Background:**

Patient informed consent is a crucial subject in preoperative care of patients before elective hand surgery, ensuring that patients have the necessary information and a comprehensive understanding to make autonomous decisions. The use of video-based informed consent systems is an innovative concept to enhance the consent process with multimedia tools. In addition to the conventional process, mostly relying on verbal communication and written documents, the video-based approach aims to present information in a standardized and visually appealing format.

**Methods:**

In this study, 33 patients were asked to watch a video on a tablet about the planned elective hand surgery after a conventional pre-treatment consultation including informed consent throughout verbal explanations and paper forms by an attending physician or resident. All patients were asked to complete a questionnaire after watching the video.

**Results:**

An overwhelming majority of participants, specifically 97.0%, stated that the video improved their understanding of the upcoming surgery. 90.9% of the participant would refer the video to other patients undergoing elective hand surgery, while 72.7% of participants indicated that they would have appreciated the opportunity to view an informational video before undergoing different types of surgeries in the past.

**Conclusion:**

The use of a video-based patient information system in elective hand surgery had a positive impact on patient education and satisfaction with the informed consent process. Therefore, it is a powerful tool in preoperative management to guarantee a standardized and educative informed consent.

**Supplementary Information:**

The online version contains supplementary material available at 10.1007/s00402-024-05291-9.

## Introduction

Informed consent serves as a foundation of patient autonomy which is based on an adequate understanding of the proposed surgical procedure including the course of action before, during and after the surgery as well as associated potential risks and benefits and treatment alternatives. To date, this process has heavily relied on verbal communication and written documents [1]. It displays a time-consuming procedure for health care providers, where time and human resources are rare in the medical field nowadays. Furthermore, there can be various obstacles in a preoperative dialogue between the surgeon and the patient which is only based on oral information and an informed consent form. The load of information can be overwhelming, the patient might be too excited or scared to ask questions or the patients are not familiar with the medial terms [2]. Data have shown that patients frequently have a restricted comprehension of the details, even after having signed a consent form [3]. Considering that information about standard surgical procedures is predominantly identical, the surgeon must repeat the same dialogue many times. Audiovisual-based supported informed consent as an additional tool is an approach to provide this information and therefore augment the quality of patient understanding so the surgeon and the patient can focus on relevant questions or personal inquiries in the dialogue [4]. The field of hand surgery is characterized by a huge variety of surgical treatment options at a complex anatomical location. However, some conditions affecting the hand are highly prevalent, especially in older patient cohorts. For instance, carpal tunnel syndrome, trigger finger and dupuytren’s contracture are very common and can be treated with standardized surgical procedures [5–7]. Therefore, the appropriate surgical interventions for those conditions are well-suited for a video-based information system. To provide a more effective and standardized informed consent we presented videos about the standardized surgical interventions of the above-mentioned conditions to the patients which were shown during the consultation on a tablet. Within the video, the hand anatomy as well as the treatment concepts, procedural details and potential risks and benefits are presented in a short amount of time. After seeing the video, the patients were asked to complete a questionnaire. The aim of this study was to elucidate the role of an additional video-based patient information in elective hand surgery.

## Material and methods

The videos were designed by e.Bavarian Health GmbH (Thieme Group). Videos about different elective hand surgery interventions (decompression of carpal tunnel, surgical therapy of trigger finger and partial fasciectomy for dupuytren’s disease) were available. For this study, only adult patients scheduled for an elective intervention who were native speakers were included. 33 patients at the department of plastic and hand surgery Erlangen (Germany) with carpal tunnel syndrome, trigger finger and dupuytren’s contracture were asked to watch the information video on a tablet at the day of preoperative consultation (length of the videos: 4:11 min to 4:48 min). Within minutes after the surgery was recommended and verbal and written consent were obtained, the video was presented to the patients in the same room where the clinical examination and the process of obtaining verbal and written consent took place. The video encompassed depictions of the disease’s pathogenesis, anatomical details, the operative setting, and the surgical procedure. All patients were then asked to complete a questionnaire in a paper form. All participants were informed that their participation was entirely voluntary, and they were free to withdraw at any point. The study was conducted over a 5-month period. In the patient’s questionnaire, the patient’s subjective opinion on the video was asked in eight questions. The patients could express their opinion on a scale reaching from 1 (I strongly agree) to 5 (I strongly disagree) (Table 1).

**Table 1:**
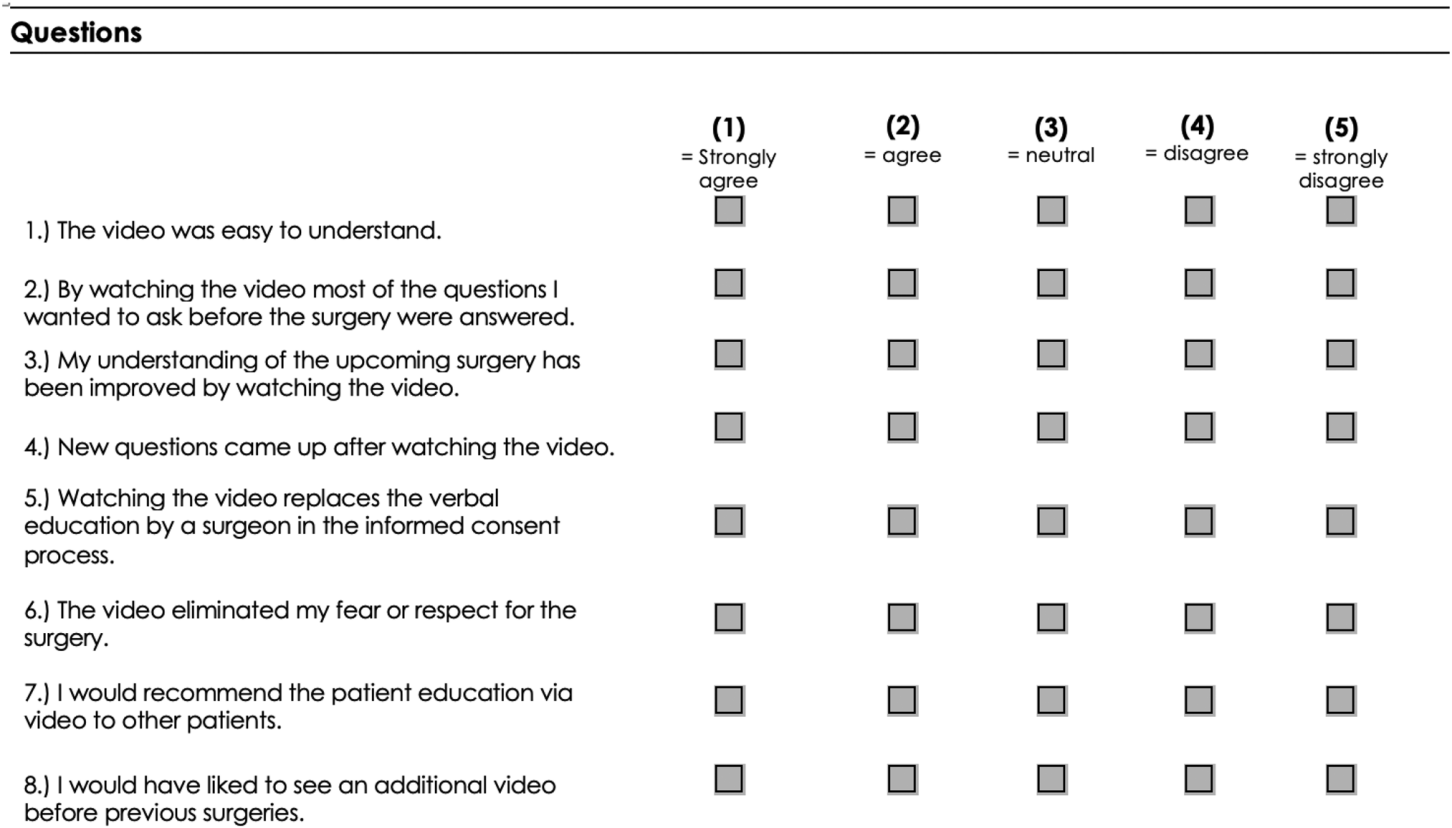
Questionnaire the patients had to fill out after watching the video(s)

## Results

**Fig. 1:**
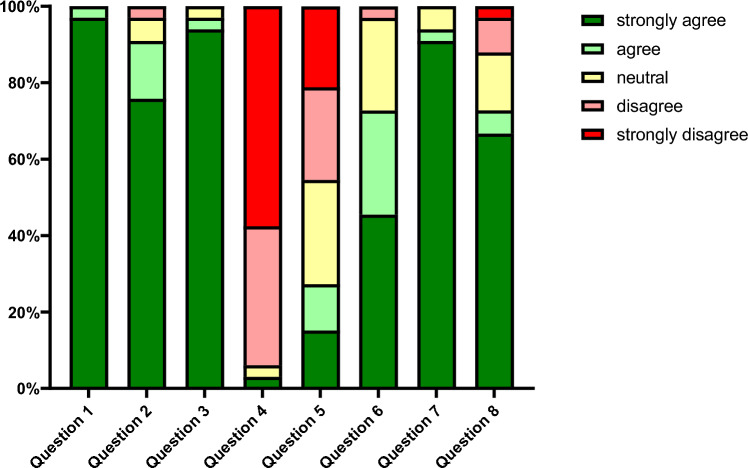
Answers of the patients on the questionnaire after watching the video(s)

Over a 5-month period, 33 participants were included in this study. The mean age of the participants was 66 years (SD = 14.89), range 30 to 87 years. 48.5% of the patients were female (*n* = 16) and 51.5% were male (*n* = 17). Fourteen patients (42.4%) underwent surgical decompression of the carpal tunnel, nine patients (27.3%) were treated for surgical therapy of trigger finger and nine patients (27.3%) underwent partial fasciectomy for dupuytren’s disease. Three patients had combined surgery for carpal tunnel syndrome and trigger finger (*n* = 2) and dupuytren’s contracture and trigger finger (*n* = 1) (Table 2). Every single participant (100%) expressed satisfaction with the clarity and comprehensibility of the video-based patient information system. A vast majority of participants, precisely 97.0%, noted that the video significantly enhanced their grasp of the forthcoming surgery. Furthermore, 90.9% of the participants expressed their intention to recommend the video to other patients scheduled for elective hand surgery. Additionally, 72.7% of participants revealed that they would have welcomed the chance to watch an informative video prior to undergoing various surgical procedures in previous experiences. Among all the patients, 93.9% verified that their questions regarding the surgical procedure had been answered by watching the video. 93.9% of the participants reported that the information program did not raise any additional questions for them. In contrast, only one patient mentioned that new questions had developed as a result of watching the video. 72.7% of the patients stated that they were calmed (15 patients “strongly agree” and 9 patients “agree”) and conversely, only one patient was rather frightened by watching the video. Eight patients were neither calmed nor frightened (eight patients “neutral”). 27.3% of the participants concurred that watching the video could serve as a substitute for the verbal informed consent with a surgeon, while 45.5% held an opposing opinion (Fig. 1).

**Table 2:** Characteristics of the 33 included patients

Patient/treatment characteristics	Values/numbers	
Median age (years)	66.2 (30–87)	
Sex		
Male	17	51.5%
Female	16	48.5%
Disease		
Carpal Tunnel syndrome	14	42.4%
Dupuytren’s disease	9	27.3%
Trigger finger	9	27.3%
Combined	3	9.1%

## Discussion

While various modern digital diagnostic tools in hand surgery have been studied extensively [8–11], there is a limited scientific evaluation of digital tools in the context of the informed consent process. Educating patients before surgery is a vital aspect of a physician's responsibility, contributing significantly to building trust between patients and the healthcare professionals. Patients expect that the information provided to them during the informed consent process is accurate, complete, and understandable. Providing clear and professional preoperative information to meet the expectations of patients can be a challenging task, particularly in an era where time is short and precious and all sorts of information is available on the internet, varying greatly in terms of quality and accuracy. Hence, not all material available on the internet will sufficiently fulfill all necessary requirements that will enable a patient to freely and competently decide whether to proceed with a surgery. To obtain genuine informed consent, it is imperative that patients receive thorough education. In recent times, this aspect has gained even greater significance due to legal requirements, particularly in the context of elective surgical procedures. It has become mandatory to adapt the intensity of the informed consent procedure to the level of medical urgency of any indication for medical treatment. Although education protocols are commonly applied, their effectiveness is hindered by variations in patient demographics, including age, gender, and socioeconomic status [12]. Watching an optional video to supplement the standard informed consent process has been proposed and studied as a tool for clinical trials, and authors have proposed that audiovisual tools might modify the traditional informed consent process for clinical studies [13]. Moreover, McNutt et al. discovered that half of the study participants spent only thirty seconds or less reading the informed consent documents [14] after providing information verbally. This suggests that incorporating a video may encourage participants or patients to engage with the topic for the duration of the video, ensuring they receive comprehensive information. The patient information videos were created to offer comprehensive information regarding upcoming surgical procedures to patients visiting our clinic preoperatively. It has been shown that the use of multimedia adjunct to the verbal informed consent process can improve the knowledge of the patients significantly [4, 15]. A systematic review found that digital technologies for informed consent were not found to negatively affect any of the outcomes, and overall, multimedia tools seem desirable [16]. The presented study involved 33 participants who completed a questionnaire after watching the video on a tablet revealed that implementing the video after the verbal explanations and the paperwork, significantly enhanced patient education within the informed consent process. The majority of the participants stated positive feedback as they would refer watching the video to other patients undergoing elective hand surgery and wished to have had the chance to watch an education video before other surgeries in the past. Additionally, after watching the video, patients noted that the video itself addressed some of the questions they had initially intended to ask the surgeon. This suggests a potential reduction in the amount of time the surgeon needs to dedicate to verbal patient education during the informed consent process. Rowbotham et al. [17] revealed that the integration of an introductory video, conventional consent language, and an interactive quiz within a tablet-based system enhances understanding of the procedures and potential risks associated with a research study. However, the study was performed for potential participants of research studies and not for patients before undergoing elective surgery. A significant limitation of this study is the absence of a control group consisting of patients solely receiving verbal and written consent. A comparative study would be more informative in demonstrating the advantages of video-based informed consent. Furthermore, it would be interesting to elucidate, whether the additional use of educational videos before surgery in the informed consent process can enhance patient knowledge not just subjectively but also objectively at different time points. In the future, further studies exploring modern communication modalities as approaches to effectively communicate information during the informed consent process should be pursued.

## Conclusion

The findings of this study undermine the potential benefits of integrating additional educational media devices into the daily clinical routine. By watching the information videos, patients can actively engage in preoperative discussions with physicians, starting from a more informed standpoint. The integration of the information videos into the clinical workflow before the planed surgery resulted in an overall increase in patient satisfaction with the informed consent process. However, it is important to emphasize that while informative tools are valuable, they cannot replace the essential personal dialogue between the surgeon and the patients, which is still the foundation for building a strong relationship between the patient and the surgeon that is crucial for achieving positive clinical outcomes.

### Supplementary Information

Below is the link to the electronic supplementary material.

Supplementary file1 (MP4 88243 kb)

## Declarations

### Supplementals

The videos used in this study were designed by e.Bavarian Health GmbH (Thieme Group).
